# The inorganic–organic hybrid material triethyl­enetetra­mmonium hexa­chlorido­rhodate(III) chloride

**DOI:** 10.1107/S1600536807067293

**Published:** 2007-12-21

**Authors:** Thomas Hahn, Walter Frank

**Affiliations:** a Institut für Anorganische Chemie und Strukturchemie, Lehrstuhl II, Heinrich-Heine-Universität Düsseldorf, Universitätsstrasse 1, 40225 Düsseldorf, Germany

## Abstract

Single crystals of the new title compound [systematic name: 1,4,7,10-tetra­zoniadecane hexa­chloridorhodate(III) chloride], [H_3_N(CH_2_)_2_NH_2_(CH_2_)_2_NH_2_(CH_2_)_2_NH_3_][RhCl_6_]Cl, were obtained from the corresponding amine and rhodium trichloride in hydro­chloric acid solution by slow crystallization under diffusion-controlled conditions at room temperature. Its solid-state structure is defined by a three-dimensional framework of numerous electrostatic-supported N—H⋯Cl hydrogen bonds between the ionic components of the compound. Within this framework, layered arrangements of the complex ions on one hand and of the protonated amines and chloride ions on the other hand, can be recognized. The octahedral hexa­chloridorhodate(III) anion resides on a 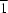 symmetry site, while the triethyl­enetetra­mmonium cation and the chloride ion both reside on twofold axes.

## Related literature

For related literature, see: Frank & Bujak (2002 ▶); Frank & Graf (2004 ▶); Frank & Reiss (1996 ▶, 1997 ▶); Frank, Reiss & Kleinwächter (1996 ▶); Gillard *et al.* (1996 ▶); Reiss (1996 ▶).
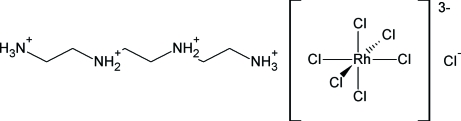


## Experimental

### 

#### Crystal data

(C_6_H_22_N_4_)[RhCl_6_]Cl
*M* *_r_* = 501.34
Monoclinic, 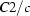

*a* = 16.8062 (13) Å
*b* = 8.7803 (8) Å
*c* = 12.3114 (11) Å
β = 108.602 (9)°
*V* = 1721.8 (3) Å^3^
*Z* = 4
Mo *K*α radiation
μ = 2.07 mm^−1^
*T* = 123 (2) K
0.6 × 0.4 × 0.2 mm

#### Data collection

Stoe IPDS-1 diffractometer
Absorption correction: analytical (Sheldrick, 1997 ▶) *T*_min_ = 0.022, *T*_max_ = 0.050
11939 measured reflections
1670 independent reflections
1518 reflections with *I* > 2σ(*I*)
*R*_int_ = 0.100

#### Refinement

*R*[*F* ^2^ > 2σ(*F* ^2^)] = 0.027
*wR*(*F* ^2^) = 0.079
*S* = 1.04
1670 reflections
96 parameters
H-atom parameters constrained
Δρ_max_ = 1.33 e Å^−3^
Δρ_min_ = −0.48 e Å^−3^

### 

Data collection: *IPDS* (Stoe & Cie (2000 ▶); cell refinement: *IPDS*; data reduction: *IPDS*; program(s) used to solve structure: *SHELXS97* (Sheldrick, 1997 ▶); program(s) used to refine structure: *SHELXL97* (Sheldrick, 1997 ▶); molecular graphics: *DIAMOND* (Brandenburg, 2006 ▶); software used to prepare material for publication: *SHELXL97* and *enCIFer* (Allen *et al.*, 2004 ▶).

## Supplementary Material

Crystal structure: contains datablocks I, global. DOI: 10.1107/S1600536807067293/gk2126sup1.cif

Structure factors: contains datablocks I. DOI: 10.1107/S1600536807067293/gk2126Isup2.hkl

Additional supplementary materials:  crystallographic information; 3D view; checkCIF report

## Figures and Tables

**Table:** 

Rh1—Cl1	2.3450 (5)
Rh1—Cl2	2.3494 (6)
Rh1—Cl3	2.3420 (6)

**Table:** 

Cl1—Rh1—Cl2	90.089 (19)
Cl1—Rh1—Cl3	89.06 (2)
Cl2—Rh1—Cl3	90.40 (2)

**Table 2:** Hydrogen-bond geometry (Å, °)

*D*—H⋯*A*	*D*—H	H⋯*A*	*D*⋯*A*	*D*—H⋯*A*
N1—H12⋯Cl1^ii^	0.90	2.34	3.213 (2)	164
N1—H13⋯Cl3	0.90	2.45	3.2195 (19)	144
N1—H11⋯Cl4	0.90	2.38	3.267 (2)	169
N2—H21⋯Cl2^iii^	0.93	2.28	3.165 (2)	159
N2—H22⋯Cl4^iv^	0.93	2.32	3.224 (2)	166

Symmetry codes: (ii) 
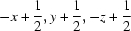
; (iii) 
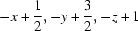
; (iv) 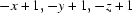.

**Fig. 1.**
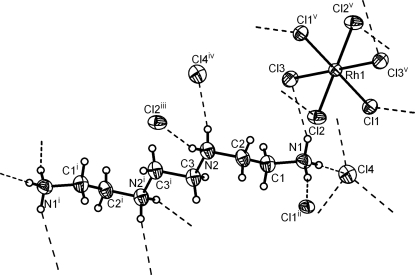
: The ionic components of triethylene tetrammonium hexachloridorhodate(III) chloride with their hydrogen bond environment. Hydrogen atoms are drawn with an arbitrary radius and displacement ellipsoids are drawn at the 50% probability level. Dashed lines indicate further hydrogen bonds establishing a three dimensional network. Hydrogen atom labels are omitted for clarity. [Symmetry codes: (i) -x + 1, -y + 2, -z + 1; (ii) -x + 1/2, y + 1/2, -z + 1/2; (iii) -x + 1/2, y + 3/2, -z + 1; (iv) -x + 1, -y + 1, -z + 1; (v) -x + 1/2, -y + 1/2, -z + 1.]

**Fig. 2.**
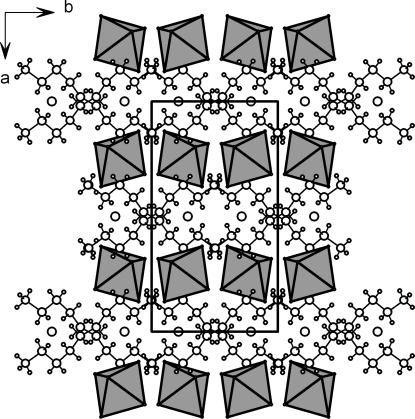
: Packing diagram, view along [001]. The [RhCl6]3- ions are arranged in layers which are separated by layers of the organic components.

**Fig. 3.**
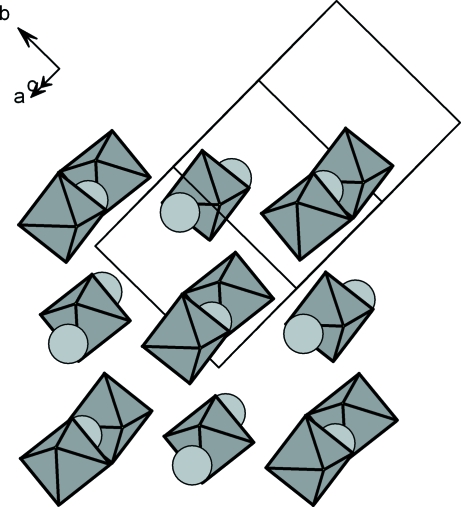
: Part of the distorted face centered cubic packing of [RhCl6]3- ions with triply charged virtual cations in the octahedral holes; note the relationship to the simple sodium chloride structure if the anions would be treated as pseudo spheres.

## supplementary crystallographic information

### Comment

As part of our research on inorganic-organic hybrid materials various alkylammonium hexahalogenidorhodates(III) have been synthesized and structurally characterized with focus on the principles of organization of the organic and inorganic components on the one hand and the hydrogen bonding networks on the other (Frank & Rei\&s, 1996; Frank & Rei\&s, 1997; Frank & Bujak, 2002; Frank & Graf, 2004). The aim of the work described in this report was to examine the structural properties of a compound extending the series of known hexachloridorhodates(III) with cations of the general formula H_3_N(CH_2_)_2_(NH_2_(CH_2_)_2_)_n_NH_3_^(n+2)+^ [n = 0 (Gillard *et al.*, 1996; Rei\&s, 1996), n = 1 (Frank *et al.*, 1996)].

A light red microcrystalline unresolvable substance is obtained in a fast precipitation reaction by mixing hydrochloric acid solutions of triethylene tetrammonium chloride (n = 2 according to the prementioned formula) and rhodium trichloride. A diffusion controlled crystallization procedure yielded single crystals of sufficient size for single-crystal structure analysis. The results of elemental analyses and spectroscopic investigations agreed with the formula [H_3_N(CH_2_)_2_(NH_2_(CH_2_)_2_)_2_NH_3_][RhCl_6_]Cl. The structure determination shows [H_3_N(CH_2_)_2_(NH_2_(CH_2_)_2_)_2_NH_3_]^4+^, [RhCl_6_]^3-^ and Cl^-^ to be present in the crystal in a ratio of 1:1:1 (Fig. 1). The [RhCl_6_]^3-^ ion has a crystallographically imposed 1 symmetry. As expected, the rhodium atom at the centre is coordinated in a nearly ideal octahedral geometry by the six chlorido ligands (Table 1). The complex ion is surrounded by four NH_3_ groups and two NH_2_ groups of altogether six triethylene tetrammonium cations. All the N–H—Cl hydrogen bonds between these cations and the chlorido ligands of the complex anion have to be considered as weak interactions (Fig. 1 and Table 2). This is indicated by N – Cl distances varying between 3.165 (2) Å and 3.267 (2) Å (with H – Cl distances from 2.28 Å to 2.45 Å) (Table 2), as well as by the IR frequencies of the N – H stretching modes. The triethylene tetrammonium cation resides on a twofold axis. Its conformation deviates substantially from the ideal all-*trans* (zigzag chain-like) arrangement. The deviation is primarily described by a torsion of 54.3 (3)° around the bond between C2 and N2, so that the cation's conformation resembles to a stretched 's'. Apart from that the bond lengths and angles are as expected. The cation has ten weak hydrogen bonds to its environment, so its hydrogen bond donor functions are completely saturated (Fig. 1). The NH_3_ group (N1) is connected to two [RhCl_6_]^3-^ octahedra by two hydrogen bonds, while the NH_2_ group (N2) is connected to a third [RhCl_6_]^3-^ octahedron, *i. e.* taking into account the site symmetry each cation contacts six octahedra. The single Cl^-^ ion (Cl4) resides on a twofold axis and is fixed by hydrogen bonds to two NH_3_ and two NH_2_ groups, which are positioned in a distorted tetrahedral arrangement (Fig. 1) and belong to four triethylene tetrammonium cations.

In total the arrangement of the ionic components defines a dense inorganic-organic three-dimensional network through extensive electrostatically supported hydrogen bonding. In principle the arrangement of the complex anions agrees well with the one described for diethylene triammonium hexachloridorhodate(III) (Frank *et al.*, 1996). The conformational flexibility of the organic cation seems to play a crucial role for the formation of a dense solid. It facilitates the organization of the cations and the single chloride anions into layers that are free of cavities. These cation/chloride layers and layers of the complex anions are stacked alternately along the crystallographic *a* axis (Fig. 2). From another point of view the arrangement of the [RhCl_6_]^3-^ ions can be regarded as a distorted face centered cubic packing, if these anions are considered to be pseudo spherical species. A structural fragment, consisting of a central chloride ion and four quarters of surrounding protonated amines, may be considered as a triply positive charged 'pseudo cation' situated in the center of the octahedral holes within the close packing of complex ions (Fig. 3).

### Experimental

Mixing hydrochloric acid solutions of triethylene tetrammonium chloride and rhodium trichloride yields a unresolvable microcrystalline powder. To obtain suitable single crystals it is necessary to slow down this precipitation reaction so that controlled growth can be accomplished. For this purpose a three chamber vessel with two lateral chambers and one central chamber, which is separated from the lateral ones by two microporous membranes was used, a setup that guarantees a slow diffusion of the components of the precipitation reaction into the central chamber. The lateral chambers were filled with 5 ml of 20% hydrochloric acid solution of rhodium trichloride and 5 ml of a saturated solution of triethylene tetrammonium in concentrated hydrochloric acid, respectively, while the central chamber contained pure concentrated hydrochloric acid. Within some days dark red brick-shaped crystals were obtained in the central chamber, that were suitable for X-ray structure analysis and single-crystal ATR-IR and Raman spectroscopy. IR data (ν, cm^-1^): 3545 (w, br), 3113 (*s*), 3083 (s, sh), 2993 (*s*) 2911 (*s*), 2845 (*s*), 2800 (*s*), 2760 (*s*), 2718 (s, sh), 2610 (m, sh), 2539 (m, sh), 2438 (w), 2390 (w), 2343 (w), 1879 (w), 1585 (*m*), 1566 (*m*), 1483 (*m*), 1457 (*m*), 1440 (*m*), 1361 (w), 1245 (w), 1295 (w), 1246 (w), 1158 (w), 1135 (w), 1072 (w), 1026 (w), 983 (w, sh), 973, (w), 892 (vw), 871 (w), 794 (vw, sh), 775 (*m*); Raman data (ν, cm^-1^): 3121 (w, sh), 2987 (w, sh), 2956 (*m*), 2882 (vw), 2824 (vw), 1566 (w), 1492 (w), 1454 (w), 1434 (w), 1401 (vw), 1336 (w), 1292 (w), 1203 (vw), 1173 (vw), 1092 (vw, sh), 1072 (w), 1013 (w), 952 (w), 824 (w), 768 (w), 513 (w), 304 (*s*), 284 (*s*), 171 (*m*), 121 (w, sh); C, H, N-analysis (501.34): C 14.46 (calc. 14.37); H 4.59 (calc. 4.42); N 10.93 (calc. 11.18) %.

### Refinement

The atomic coordinates of hydrogen atoms in idealized positions were included in the refinement in riding model approximation. C–H and N–H distances for the CH_2_, NH_2_ and NH_3_ groups were allowed to refine, the same shifts being applied along the C–H and N–H bonds of a group, respectively, and in additon the torsion angle of the NH_3_ group was allowed to refine freely. *U*_iso_(H) was set to 1.2 *U*_eq_(carrier atom) for the CH_2_ groups. A common *U*_iso_ value was refined for the hydrogen atoms of the NH_2_ and the NH_3_ group, respectively. Only one significant electron-density maximum (1.33 e Å^-3^ at 0.87 Å from Rh1) was found in the final difference Fourier map.

### Crystal data

**Table:**

(C_6_H_22_N_4_)[RhCl_6_]Cl	*F*_000_ = 1000
*M**_r_* = 501.34	*D*_x_ = 1.934 Mg m^−^^3^
Monoclinic, *C*2/*c*	Mo *K*α radiation λ = 0.71073 Å
*a* = 16.8062 (13) Å	Cell parameters from 7998 reflections
*b* = 8.7803 (8) Å	θ = 5.1–51.8º
*c* = 12.3114 (11) Å	µ = 2.07 mm^−^^1^
β = 108.602 (9)º	*T* = 123 (2) K
*V* = 1721.8 (3) Å^3^	Brick shaped, dark red
*Z* = 4	0.6 × 0.4 × 0.2 mm

### Data collection

**Table:**

Stoe IPDS-1 diffractometer	1670 independent reflections
Radiation source: fine-focus sealed tube	1518 reflections with *I* > 2σ(*I*)
Monochromator: graphite	*R*_int_ = 0.100
Detector resolution: 0 pixels mm^-1^	θ_max_ = 26.0º
*T* = 123(2) K	θ_min_ = 2.7º
φ scans	*h* = −20→20
Absorption correction: analytical(Sheldrick, 1997)	*k* = −10→10
*T*_min_ = 0.022, *T*_max_ = 0.050	*l* = −15→15
11939 measured reflections	

### Refinement

**Table:**

Refinement on *F*^2^	Secondary atom site location: difference Fourier map
Least-squares matrix: full	Hydrogen site location: inferred from neighbouring sites
*R*[*F*^2^ > 2σ(*F*^2^)] = 0.027	H-atom parameters constrained
*wR*(*F*^2^) = 0.079	*w* = 1/[σ^2^(*F*_o_^2^) + (0.0533*P*)^2^ + 0.1116*P*] where *P* = (*F*_o_^2^ + 2*F*_c_^2^)/3
*S* = 1.04	(Δ/σ)_max_ < 0.001
1670 reflections	Δρ_max_ = 1.33 e Å^−^^3^
96 parameters	Δρ_min_ = −0.48 e Å^−^^3^
Primary atom site location: structure-invariant direct methods	Extinction correction: none

### Special details

**Table:**

Geometry. All e.s.d.'s (except the e.s.d. in the dihedral angle between two l.s. planes) are estimated using the full covariance matrix. The cell e.s.d.'s are taken into account individually in the estimation of e.s.d.'s in distances, angles and torsion angles; correlations between e.s.d.'s in cell parameters are only used when they are defined by crystal symmetry. An approximate (isotropic) treatment of cell e.s.d.'s is used for estimating e.s.d.'s involving l.s. planes.
Refinement. Refinement of *F*^2^ against ALL reflections. The weighted *R*-factor *wR* and goodness of fit *S* are based on *F*^2^, conventional *R*-factors *R* are based on *F*, with *F* set to zero for negative *F*^2^. The threshold expression of *F*^2^ > σ(*F*^2^) is used only for calculating *R*-factors(gt) *etc*. and is not relevant to the choice of reflections for refinement. *R*-factors based on *F*^2^ are statistically about twice as large as those based on *F*, and *R*- factors based on ALL data will be even larger.

### Fractional atomic coordinates and isotropic or equivalent isotropic displacement parameters (Å^2^)

**Table:**

	*x*	*y*	*z*	*U*_iso_*/*U*_eq_	
Rh1	0.2500	0.2500	0.5000	0.01442 (13)	
Cl1	0.30357 (4)	0.15203 (6)	0.36024 (4)	0.01911 (16)	
Cl2	0.18197 (4)	0.44496 (6)	0.37375 (5)	0.02252 (17)	
Cl3	0.37037 (4)	0.40149 (6)	0.56865 (4)	0.02213 (16)	
Cl4	0.5000	0.28717 (11)	0.2500	0.0277 (2)	
N1	0.36274 (14)	0.5023 (2)	0.31408 (16)	0.0223 (4)	
H11	0.3943	0.4372	0.2892	0.036*	
H12	0.3197	0.5347	0.2544	0.036*	
H13	0.3428	0.4549	0.3646	0.036*	
N2	0.41689 (14)	0.8552 (2)	0.49770 (18)	0.0229 (4)	
H21	0.3832	0.9274	0.5171	0.040*	
H22	0.4446	0.8006	0.5637	0.040*	
C1	0.41463 (17)	0.6344 (3)	0.3702 (2)	0.0228 (5)	
H31	0.4361	0.6803	0.3204	0.027*	
H32	0.4576	0.6022	0.4304	0.027*	
C2	0.3615 (2)	0.7477 (2)	0.4109 (3)	0.0238 (6)	
H41	0.3273	0.8035	0.3478	0.029*	
H42	0.3262	0.6947	0.4440	0.029*	
C3	0.48081 (16)	0.9362 (3)	0.4577 (2)	0.0229 (5)	
H51	0.4548	0.9776	0.3828	0.027*	
H52	0.5236	0.8665	0.4539	0.027*	

### Atomic displacement parameters (Å^2^)

**Table:**

	*U*^11^	*U*^22^	*U*^33^	*U*^12^	*U*^13^	*U*^23^
Rh1	0.0143 (2)	0.01504 (18)	0.01519 (18)	0.00114 (8)	0.00653 (13)	0.00025 (8)
Cl1	0.0210 (3)	0.0198 (3)	0.0190 (3)	0.0017 (2)	0.0099 (2)	−0.00171 (19)
Cl2	0.0247 (4)	0.0230 (3)	0.0223 (3)	0.0078 (2)	0.0109 (2)	0.0065 (2)
Cl3	0.0205 (3)	0.0269 (3)	0.0200 (3)	−0.0064 (2)	0.0079 (2)	−0.0027 (2)
Cl4	0.0256 (5)	0.0257 (4)	0.0321 (5)	0.000	0.0094 (4)	0.000
N1	0.0258 (12)	0.0213 (10)	0.0208 (10)	−0.0014 (9)	0.0087 (8)	0.0009 (8)
N2	0.0223 (12)	0.0204 (10)	0.0268 (10)	0.0005 (9)	0.0089 (9)	−0.0026 (8)
C1	0.0220 (14)	0.0225 (11)	0.0243 (11)	−0.0018 (10)	0.0080 (10)	−0.0009 (9)
C2	0.0236 (17)	0.0210 (14)	0.0273 (14)	−0.0005 (9)	0.0087 (12)	−0.0009 (8)
C3	0.0200 (13)	0.0207 (11)	0.0294 (12)	0.0021 (10)	0.0100 (10)	−0.0010 (10)

### Geometric parameters (Å, °)

**Table:**

Rh1—Cl1	2.3450 (5)	N1—H13	0.8970
Rh1—Cl2	2.3494 (6)	N2—H21	0.9301
Rh1—Cl3	2.3420 (6)	N2—H22	0.9301
N1—C1	1.484 (3)	C1—H31	0.9002
N2—C2	1.505 (3)	C1—H32	0.9002
N2—C3	1.497 (3)	C2—H41	0.9429
C1—C2	1.524 (4)	C2—H42	0.9429
C3—C3^i^	1.527 (5)	C3—H51	0.9563
N1—H11	0.8970	C3—H52	0.9563
N1—H12	0.8970		
			
Cl1—Rh1—Cl2	90.089 (19)	H21—N2—H22	107.6
Cl1—Rh1—Cl3	89.06 (2)	N1—C1—H31	109.6
Cl2—Rh1—Cl3	90.40 (2)	C2—C1—H31	109.6
N1—C1—C2	110.2 (2)	N1—C1—H32	109.6
C1—C2—N2	110.3 (2)	C2—C1—H32	109.6
C2—N2—C3	114.2 (2)	H31—C1—H32	108.1
N2—C3—C3^i^	108.4 (2)	N2—C2—H41	109.6
C1—N1—H11	109.5	C1—C2—H41	109.6
C1—N1—H12	109.5	N2—C2—H42	109.6
H11—N1—H12	109.5	C1—C2—H42	109.6
C1—N1—H13	109.5	H41—C2—H42	108.1
H11—N1—H13	109.5	N2—C3—H51	110.0
H12—N1—H13	109.5	C3^i^—C3—H51	110.0
C3—N2—H21	108.7	N2—C3—H52	110.0
C2—N2—H21	108.7	C3^i^—C3—H52	110.0
C3—N2—H22	108.7	H51—C3—H52	108.4
C2—N2—H22	108.7		
			
N1—C1—C2—N2	162.70 (19)	C2—N2—C3—C3^i^	168.2 (2)
C3—N2—C2—C1	54.3 (3)		

Symmetry codes: (i) −*x*+1, −*y*+2, −*z*+1.

### Hydrogen-bond geometry (Å, °)

**Table:**

*D*—H···*A*	*D*—H	H···*A*	*D*···*A*	*D*—H···*A*
N1—H12···Cl1^ii^	0.90	2.34	3.213 (2)	164
N1—H13···Cl3	0.90	2.45	3.2195 (19)	144
N1—H11···Cl4	0.90	2.38	3.267 (2)	169
N2—H21···Cl2^iii^	0.93	2.28	3.165 (2)	159
N2—H22···Cl4^iv^	0.93	2.32	3.224 (2)	166

Symmetry codes: (ii) −*x*+1/2, *y*+1/2, −*z*+1/2; (iii) −*x*+1/2, −*y*+3/2, −*z*+1; (iv) −*x*+1, −*y*+1, −*z*+1.

## References

[bb1] Allen, F. H., Johnson, O., Shields, G. P., Smith, B. R. & Towler, M. (2004). *J. Appl. Cryst.* **37**, 335–338.

[bb2] Brandenburg, K. (2006). *DIAMOND* Version 3.1b. Crystal Impact GbR, Bonn, Germany.

[bb3] Frank, W. & Bujak, M. (2002). *Z. Naturforsch. Teil B*, **57**, 1391–1400.

[bb4] Frank, W. & Graf, J. (2004). *Z. Anorg. Allg. Chem.* **630**, 1894–1902.

[bb5] Frank, W. & Reiss, G. J. (1996). *Chem. Ber.* **129**, 1355–1359.

[bb6] Frank, W. & Reiss, G. J. (1997). *Inorg. Chem.* **36**, 4593–4595. 10.1021/ic970337a 11670126

[bb7] Frank, W., Reiss, G. J. & Kleinwächter, I. (1996). *Z. Anorg. Allg. Chem.* **622**, 729–733.

[bb8] Gillard, R. D., Hibbs, D. E., Holland, C., Hursthouse, M. B., Malik, A. & Sykara, G. (1996). *Polyhedron*, **15**, 225–232.

[bb9] Reiss, G. J. (1996). Thesis, University of Kaiserslautern, Germany.

[bb10] Sheldrick, G. M. (1997). *SHELXS97* and *SHELXL97* University of Göttingen, Germany.

[bb11] Stoe & Cie (2000). *IPDS.* Version 2.93. Stoe & Cie, Darmstadt, Germany.

